# IgE sensitisation in relation to flow-independent nitric oxide exchange parameters

**DOI:** 10.1186/1465-9921-7-92

**Published:** 2006-06-20

**Authors:** Andrei Malinovschi, Christer Janson, Thomas Holmkvist, Dan Norbäck, Pekka Meriläinen, Marieann Högman

**Affiliations:** 1 Department of Medical Cell Biology: Integrative Physiology, Uppsala University, Uppsala, Sweden; 2 Asthma and Allergy Research Centre, Uppsala University, Uppsala, Sweden; 3 Department of Medical Sciences: Respiratory Medicine and Allergology, Uppsala University, Uppsala, Sweden; 4 Department of Medical Sciences: Occupational and Environmental Medicine, Uppsala University, Uppsala, Sweden; 5 Department of Engineering Physics and Mathematics, Helsinki University of Technology, Helsinki, Finland; 6 Department of Caring Sciences and Sociology; Section of Medical Science, University of Gävle, Gävle, Sweden

## Abstract

**Background:**

A positive association between IgE sensitisation and exhaled NO levels has been found in several studies, but there are no reports on the compartment of the lung that is responsible for the increase in exhaled NO levels seen in IgE-sensitised subjects.

**Methods:**

The present study comprised 288 adult subjects from the European Community Respiratory Health Survey II who were investigated in terms of lung function, IgE sensitisation (sum of specific IgE), smoking history and presence of rhinitis and asthma. Mean airway tissue concentration of NO (Caw_NO_), airway transfer factor for NO (Daw_NO_), mean alveolar concentration of NO (Calv_NO_) and fractional exhaled concentration of NO at a flow rate of 50 mL s^-1 ^(FE_NO 0.05_) were determined using the extended NO analysis.

**Results:**

IgE-sensitised subjects had higher levels (geometric mean) of FE_NO 0.05 _(24.9 vs. 17.3 ppb) (p < 0.001), Daw_NO _(10.5 vs. 8 mL s^-1^) (p = 0.02) and Caw_NO _(124 vs. 107 ppb) (p < 0.001) and positive correlations were found between the sum of specific IgE and FE_NO 0.05_, Caw_NO _and Daw_NO _levels (p < 0.001 for all correlations). Sensitisation to cat allergen was the major determinant of exhaled NO when adjusting for type of sensitisation. Rhinitis and asthma were not associated with the increase in exhaled NO variables after adjusting for the degree of IgE sensitisation.

**Conclusion:**

The presence of IgE sensitisation and the degree of allergic sensitisation were related to the increase in airway NO transfer factor and the increase in NO concentration in the airway wall. Sensitisation to cat allergen was related to the highest increases in exhaled NO parameters. Our data suggest that exhaled NO is more a specific marker of allergic inflammation than a marker of asthma or rhinitis.

## Background

An increase in exhaled nitric oxide (NO) levels due to IgE sensitisation was first observed in laboratory animal allergy [1] and asymptomatic atopic subjects[2]. A positive association between exhaled NO levels and the degree of IgE sensitisation has been found both in children [3-6] and in the adult population [7]. In these investigations, the degree of IgE sensitisation has been measured as the number of positive allergens in skin prick testing[3,6,7] or the sum of the weal diameters for the investigated allergens (skin prick test index) in children[4,5]. Recently, calculating the sum of specific IgE levels against the allergens of interest has been proposed as an alternative method for measuring the degree of IgE sensitisation[8,9].

The mechanism behind the increased levels of exhaled NO in IgE-sensitised subjects is not fully understood. Atopic, non-asthmatic subjects often have a subclinical airway inflammation[10]. This eosinophilic inflammation causes lung tissue damage followed by the release of cytokines and the stimulation of inducible nitric oxide synthase (iNOS). Exposure to allergens might also stimulate bronchial epithelium iNOS [11] and increase exhaled NO levels. It has also been proposed that there is a common gene that regulates iNOS and atopic activity [12]. The increase in epithelial iNOS activity probably explains the increase in NO levels in IgE-sensitised subjects, since epithelial iNOS activity has been shown to be the main determinant of FE_NO _in humans[13].

It is possible to obtain a greater insight into the two NO-producing compartments, the airways and alveoli, by modelling NO exchange dynamics. These models are characterised by two or three NO flow-independent parameters, depending on the model [14]. There are no studies which directly analyse the effects of IgE sensitisation on NO flow-independent parameters. Subjects with allergic asthma [15-17] have been found to have increased NO concentrations in the airway wall and a higher NO airway transfer factor than healthy controls, while subjects with allergic rhinitis[17] have been found to have a higher NO airway transfer factor. These previous studies did not include subjects with non-allergic asthma or rhinitis and it is therefore not possible to understand the impact of IgE sensitisation alone on NO flow-independent parameters from the studies published so far.

The aim of the present investigation was to study where the NO responsible for the increase in the levels of exhaled NO seen in IgE-sensitised subjects comes from.

## Methods

### Population

The European Community Respiratory Health Survey (ECRHS) is an international multi-centre study of asthma and allergy. The first part, ECRHS I, was conducted in 1990–1994 and the follow-up study, ECRHS II, in 1999–2001. The design of ECRHS I and ECRHS II has been published in detail[18,19]. Each participant was sent a brief questionnaire (Stage 1) and, from those who responded, a random sample was invited to undergo a more detailed clinical examination (Stage 2). A "symptomatic sample" consisting of additional subjects who reported symptoms of waking with shortness of breath, asthma attacks or using asthma medication in Stage 1 was also studied. In ECRHS II, subjects who had participated in Stage 2 of ECRHS I were invited to participate in a follow-up study. Subjects answered a standardised questionnaire administered by trained interviewers and underwent lung function tests and blood tests.

Of the 823 subjects who participated in Stage 2 of the ECRHS in Uppsala, 679 were re-investigated nine years later (1999–2000) in the ECRHS II[19]. Of these, 489 were seen at the hospital for a clinical examination, lung function tests and blood tests, while the remaining subjects only participated in a telephone survey, usually because they had moved outside the study area between the two surveys. Of the subjects who attended the clinical examination, 288 (59%) were also willing to perform exhaled NO measurements at multiple flow rates.

### Questionnaires

The ECRHS II main questionnaire [19] was used to obtain information about allergic symptoms, exposure to investigated allergens and smoking history.

### Measurements of exhaled NO

The NO measurements were performed according to American Thoracic Society (ATS) recommendations, apart from the use of three additional flows (5, 100 and 500 mL s^-1^) and no vital capacity manoeuvre, as a deep breath with slow inhalation was found to be sufficient[20].

The system used for NO measurements was a computer-based, single-breath NO system from Nitrograf AB, Hässelby, Sweden, which used a chemiluminescence analyser (Sievers NOA 280, Sievers, Boulder, CO, USA). The system was calibrated using a mixture of 460 ppb NO in nitrogen (AGA AB, Lidingö, Sweden) and the zero was set by feeding synthetic air (AGA AB) into a 2 L canister filled with Purafil II chemisorbant with purakol (Lindair AB, Ljusne, Sweden). The flow sensor was calibrated in the range of 0–0.6 L sec^-1 ^(Dry Cal DC-2 flow calibrator, BIOS International, Pompton Plains, NJ, USA). Checks of the calibration and flow rate of the sampling system were made on a daily basis and the zero was controlled before each measurement. The expiratory pressure for all subjects was between 5 and 20 cm H_2_O in order to exclude a NO contribution from the nasal cavity. A mean value of three breaths (or two if the NO concentrations were identical from the two breaths) was used for statistical analysis.

### Application of the extended NO analysis

The extended NO analysis has previously been described and validated[17]. Using the values of fractional exhaled nitric oxide (FE_NO_) collected at three different flow rates (5, 100 and 500 mL s^-1^) and an iteration algorithm, it calculates the three flow-independent NO parameters confined to the two compartments: conducting airways, which are characterised by the mean airway tissue concentration of NO (Caw_NO_) and NO airway transfer factor (Daw_NO_), and alveoli, characterised by a mean alveolar tissue concentration of NO (Calv_NO_). The fractional exhaled nitric oxide value at a flow rate of 50 mL s^-1 ^(FE_NO 0.05_) was used as a measure of the overall exhaled NO concentration. We chose to use the FE_NO 0.05 _value in order to have a reference value for the other studies and to comply with ATS recommendations [21].

### Lung function

Forced expiratory volume in one second (FEV_1_) was measured using a dry rolling seal spirometer system (Sensor Medics 2130, Sensor Medics, Anaheim, California, USA). Up to five technically acceptable blows were determined. The ATS recommendations were followed[22]. The predicted values for forced expiratory volume in one second (FEV_1_) were calculated on the basis of the European Coal and Steel Union reference values[23].

### IgE sensitisation

Blood samples were collected for the measurement of total and specific serum IgE using the Pharmacia CAP System (Pharmacia Diagnostics, Uppsala, Sweden). Specific IgE was measured against *Dermatophagoides pteronyssinus*, cat, timothy grass and *Cladosporium herbarum*. The detection of specific IgE of ≥ 0.35 kU/l was used as a definition of sensitisation to a specific allergen. ***IgE sensitisation ***was defined as sensitisation to at least one of the investigated allergens.

The ***degree of sensitisation ***was defined in two ways: either based on the number of allergens to which one person was sensitised or using a continuous variable – the sum of specific IgE – that has been defined as the sum of the specific IgE titres for the investigated allergens.

A titre below the detection level (<0.35 kU/L) was arbitrarily given the value 0.17 kU/L.

### Diagnosis of asthma and rhinitis

A positive diagnosis of ***rhinitis ***was made in the individuals who answered positively to the question "Do you have any nasal allergies, including hay fever?".

A person was recorded as having ***asthma ***if he/she had ever been diagnosed with asthma and had an asthma attack or one of the following symptoms during the last 12 months: nocturnal chest tightness, attack of shortness of breath, chest wheezing or whistling[24].

### Smoking history

Information on smoking history was retrieved from the main questionnaire, in ECRHS II. Those who answered "yes" to the lead question ("Have you ever smoked for as long as a year?") were classified as ***ex-smokers ***and ***current smokers ***based on a negative/positive answer to the question regarding current consumption ("Do you now smoke, as of one month ago?").

### Statistical methods

Statistical analyses were performed using STATA 8.0 software (Stata Corp., 2001, Texas, USA). NO values, specific IgE titres, the sum of specific IgE titres and total IgE titres were log transformed before analysis. Chi-squared test and unpaired t-test were used when comparing subjects who performed NO measurements and the rest of the subjects in ECRHS II. Unpaired t-test was used when comparing exhaled NO and NO flow-independent parameters between sensitised and non-sensitised subjects. Linear regression was used in the bivariate analyses to analyse the correlation between the degree of IgE sensitisation and exhaled NO variables. Multiple linear regression was used when analysing the effect of different explanatory variables on exhaled NO and the NO flow-independent parameters. These models always included age, gender, height, FEV_1 _and smoking history. A p-value of < 0.05 was considered statistically significant.

### Ethics

All the subjects gave their permission for the utilisation of personal data for the purpose of this study. The study was approved by the Ethics Committee at the Medical Faculty at Uppsala University.

## Results

The subjects who underwent exhaled NO measurements (Table 1) did not differ from the other ECRHS II participants in terms of IgE sensitisation and smoking history. However, there were differences between the investigated group and the rest of the subjects in terms of age (43 vs. 41 years, p < 0.0001) and gender distribution (44.8% women vs. 53.8% women, p = 0.01).

**Table 1:** Characteristics of study population

**Age (mean (range))**	43 (29–54)			
**Gender (M/F)**	159 (55%)/129 (45%)			
Smoking status*	**Non-smokers**		**Smokers**	
	237 (83%)		49 (17%)	

**Atopy status**	**Non-atopic**		**Atopic**	
	177 (61%)		111 (39%)	
				

**Allergen**	**Cat**	**Timothy**	**Mite**	***Cladosporium***

**Sensitised subjects**	75 (26%)	65 (23%)	26 (10%)	8 (3%)
**Titres of sIgE(kU/L)**	1.47 (0.98, 2.20)	1.23 (0.86, 1.76)	1.71 (0.94, 3.12)	1.95 (0.66, 5.74)
				

**Respiratory disease**^**#**^	**None**	**Asthma +/- rhinitis**		**Only rhinitis**

**All subjects**	148 (52%)	57 (20%)		80 (28%)
**Sensitized subjects**	22 (15%)	42 (74%)		46 (58%)
**On daily therapy**^**§**^	1 (1%)	15 (26%)		1 (1%)

All results expressed as N(%) or geometric mean (95% CI) with the exception of age.

* Smoking status was not recorded for 2 subjects.

^# ^Information on respiratory disease was not available for 3 subjects.

^§ ^with inhaled corticosteroids or oral antileukotrienes

### IgE sensitisation

IgE sensitisation was associated with higher levels of FE_NO 0.05_, Caw_NO _and Daw_NO _(Table 2). The associations with FE_NO 0.05 _and Daw_NO _remained significant after adjusting for potential confounders (gender, height, age, smoking history and FEV_1_) (Table 2).

**Table 2:** Exhaled NO levels (ppb, geometric mean (95% CI)) in non-sensitised and sensitised subjects

	**Non sensitised (n = 177)**	**Sensitised (n = 111)**	**p-value**	**p-value after adjustments**
**FE**_**NO 0.05 **_**(ppb)**	17.3 (16.0–18.7)	24.9 (21.9–28.4)	<0.0001	<0.001
**Caw**_**NO **_**(ppb)**	107 (98.5–115)	124 (111–139)	0.02	0.059
**Daw**_**NO **_**(mL s^**-1**^)**	8.00 (7.35–8.71)	10.5 (9.52–11.6)	0.0001	0.002
**Calv**_**NO **_**(ppb)**	1.28 (1.12–1.46)	1.41 (1.22–1.62)	0.35	0.23

### Degree of sensitisation

**Figure 1:**
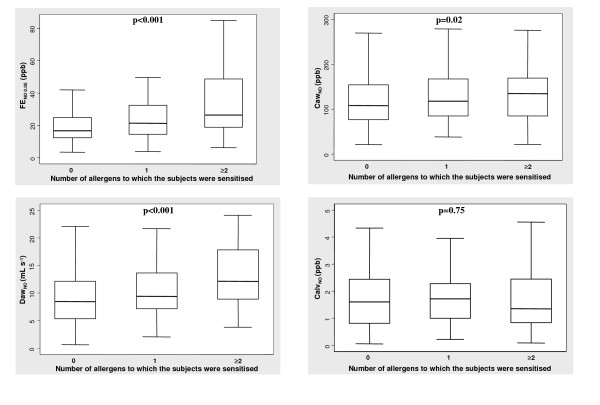
**Exhaled NO and flow-independent airway NO exchange parameters in relation to the degree of sensitisation, assessed by the number of allergens to which one person was sensitised**. Box-plot explanation: upper horizontal line of box, 75^th ^percentile; lower horizontal line of box, 25^th ^percentile; horizontal bar within box, median; upper horizontal bar outside box, upper adjacent value; lower horizontal bar outside box, lower adjacent value

FE_NO 0.05 _and the airway NO flow-independent parameters increased with the number of allergens to which the subject was sensitised (p for trend <0.001 for FE_NO 0.05_, p = 0.02 for Caw_NO _and p < 0.001 for Daw_NO_), while there was no significant correlation between the number of allergens and Calv_NO _(p = 0.75) (Figure 1). These associations remained statistically significant after adjusting for gender, height, age, smoking history and FEV_1_.

**Figure 2:**
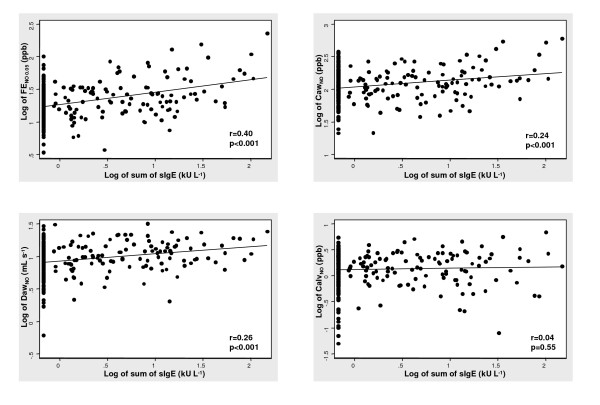
Exhaled NO and flow-independent airway NO exchange parameters in relation to degree of sensitisation, quantified by the sum of specific IgE titres against investigated allergens.

Significant correlations were found between the sum of specific IgE titres and FE_NO 0.05 _(r = 0.40, p < 0.0001), Caw_NO _(r = 0.24, p < 0.0001) and Daw_NO _(r = 0.26, p < 0.0001) respectively (Figure 2). All these relationships were maintained after adjusting for the possible confounders (p < 0.001). No correlation was found with Calv_NO _(p = 0.55). Total IgE was also significantly associated with the exhaled NO variables, but the correlation was lower than that found for the sum of specific IgE (FE_NO 0.05 _(r = 0.23, p < 0.001), Caw_NO _(r = 0.15, p = 0.01) and Daw_NO _(r = 0.13, p = 0.03)).

### Sensitisation to specific allergens

**Figure 3:**
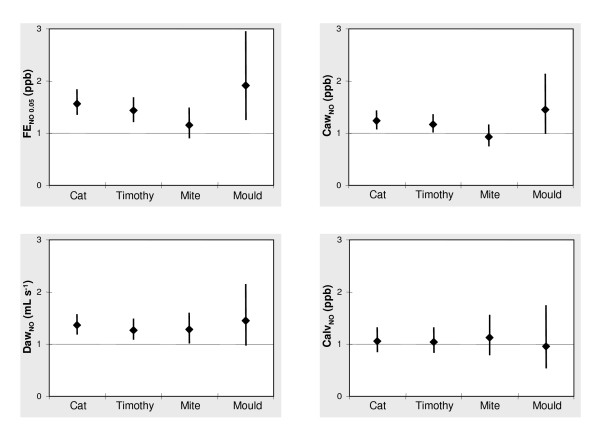
Exhaled NO ratio and flow-independent airway NO exchange parameters ratio between sensitised and non-sensitised subjects to a specific allergen (ratio of geometric means (95% CI)).

Sensitisation to all allergens except mite was associated with higher FE_NO 0.05 _levels (Figure 3). There was, for example, an increase of 57% in FE_NO 0.05 _in the cat-allergen-sensitised subjects compared with cat allergen non-sensitised subjects. Sensitisation to cat allergen was also associated with higher Caw_NO _and Daw_NO _levels, while sensitisation to timothy was associated with higher Caw_NO _levels and sensitisation to mite with higher Daw_NO _levels (Figure 3).

Significant correlations were found between the IgE titre against cat allergen and FE_NO 0.05 _(r = 0.39, p < 0.001), Caw_NO _(r = 0.26, p < 0.001) and Daw_NO _(r = 0.23, p < 0.001) respectively (Table 3). A significant positive correlation between exhaled NO and the IgE titre against timothy was also found (FE_NO 0.05 _(r = 0.27, p < 0.001), Caw_NO _(r = 0.16, p = 0.008) and Daw_NO _(r = 0.18, p = 0.002)), while the IgE titre against mite correlated positively with FE_NO 0.05 _(r = 0.16, p = 0.007) and Daw_NO _(r = 0.16, p = 0.005).

**Table 3:** Association between IgE titres against specific allergens and exhaled NO levels after adjustments for IgE titres of other allergens, gender, height, age, smoking history and FEV_1_

	**Cat**	**Timothy**	**Mite**	**Mould**
**FE**_**NO 0.05**_	0.14 (0.10, 0.19)	0.02 (-0.03, 0.07)	0.06 (-0.03, 0.14)	-0.06 (-0.21, 0.09)
**Caw**_**NO**_	0.10 (0.05, 0.15)	0.01 (-0.04, 0.05)	-0.01 (-0.09, 0.07)	-0.04 (-0.18, 0.11)
**Daw**_**NO**_	0.06 (0.02, 0.11)	0.01 (-0.03, 0.06)	0.08 (-0.004, 0.16)	-0.03 (-0.18, 0.12)
**Calv**_**NO**_	0.02 (-0.05, 0.10)	-0.01 (-0.08, 0.06)	0.03 (-0.10, 0.15)	-0.03 (-0.26, 0.19)

The effect estimate was log transformed and presented with a 95% confidence interval.

The association between the IgE titre against cat and the exhaled NO variables remained significant after adjusting for the specific IgE levels against the other allergens, gender, height, age, smoking history and FEV_1 _(Table 3).

No significant difference in exhaled NO levels was found between cat-sensitised subjects who did not have a cat (n = 63) and those that had a cat (n = 12): FE_NO 0.05 _27.5 (23.2–32.6) vs 30.0 (17.1–52.4) ppb (p = 0.70), Caw_NO _132 (115–153) vs 135 (77.9–234) ppb (p = 0.92) and Daw_NO _11.0 (9.7–12.5) vs 11.8 (8.9–15.7) mL s^-1 ^(p = 0.66).

### Asthma and rhinitis

Subjects with rhinitis (n = 79) and asthma (n = 42) had higher FE_NO 0.05 _levels (22.3 and 27.8 vs. 17.4 ppb) (p = 0.01 and p < 0.001 respectively) than subjects without a respiratory disease (n = 147). Subjects with asthma also had higher Daw_NO _(10.8 vs. 8.1 mL s^-1^) (p = 0.009) and a trend towards increased Caw_NO _(135 vs 107 ppb) (p = 0.057) than subjects without a respiratory disease, while the subjects with rhinitis displayed a trend towards increased Daw_NO _(9.6 vs. 8.1 mL s^-1^) (p = 0.07). No differences were found regarding Calv_NO _between the three investigated groups (1.42 and 1.48 vs. 1.24 ppb) (p > 0.05). Subjects using inhaled corticosteroids or oral anti-leukotrienes (n = 17) on a daily basis were excluded from this analysis.

The association between rhinitis and asthma and FE_NO 0.05 _remained significant after adjusting for gender, age, height, smoking history and lung function (FEV_1_). Both having rhinitis and having asthma were associated with increases in Daw_NO _(Table 4). All these associations became statistically non-significant after adjusting for the degree of sensitisation (Table 4). The degree of sensitisation was related to exhaled NO and airway flow-independent NO exchange parameters even after adjustment for rhinitis and asthma (Table 4).

**Table 4:** Association between exhaled NO and airway flow-independent NO exchange parameters and rhinitis, asthma and the sum of specific IgE with and without adjustment for the variables in the table.

		Unadjusted*	Adjusted **
**Rhinitis**	FE_NO 0.05_	0.13 (0.06, 0.19)	0.05 (-0.02, 0.012)
	Caw_NO_	0.06 (-0.003, 0.12)	0.01 (-0.06, 0.08)
	Daw_NO_	0.08 (0.01, 0.14)	0.04 (-0.03, 0.11)

**Asthma**	FE_NO 0.05_	0.18 (0.09, 0.26)	0.07 (-0.03, 0.16)
	Caw_NO_	0.08 (-0.0004, 0.16)	0.01 (-0.08, 0.10)
	Daw_NO_	0.11 (0.03, 0.20)	0.06 (-0.03, 0.16)

**Sum of specific IgE**	FE_NO 0.05_	0.18 (0.13, 0.23)	0.15 (0.08, 0.21)
	Caw_NO_	0.10 (0.05, 0.15)	0.09 (0.03, 0.16)
	Daw_NO_	0.10 (0.05, 0.15)	0.07 (0.01, 0.13)

The effect estimate was log transformed and presented with a 95% confidence interval.

Subjects using inhaled corticosteroids or oral anti-leukotrienes on a daily basis were excluded from the analysis.

* Adjusted for gender, age, height, FEV_1 _and smoking history but not adjusted for the variables in the table

** Adjusted both for the variables in the table and gender, age, height, FEV_1 _and smoking history

## Discussion

The main finding in the present study was that IgE sensitisation was related to an increase in airway NO transfer factor and an increase in NO concentration in the airway wall. These NO variables were also positively related to the degree of allergic sensitisation. Sensitisation to cat allergen was the allergen sensitisation related to the highest increases in exhaled NO levels. The presence of asthma or rhinitis was not related to the exhaled NO variables after adjusting for the degree of sensitisation, suggesting that exhaled NO is probably more a specific marker of allergic inflammation than a marker of asthmatic inflammation.

The present study is the first to analyse the relationship between IgE sensitisation and NO flow-independent parameters. The increased Caw_NO _in IgE-sensitised subjects probably reflects an increase in NO production due to the induction of iNOS in the airway epithelial cells, the main determinant of NO concentration in exhaled breath according to a recent study[13]. Other possible mechanisms include an increase in S-nitrosoglutathione (GSNO) reductase activity with an increase in NO release through the breakdown of S-nitrosothiols [25]. Another possible explanation is that allergic asthma is associated with a lower pH in the airway fluid [26], which may increase NO release through the protonation of nitrite and the production of nitrous acid. The increased Daw_NO _observed in IgE-sensitised subjects may be partly related to the inflammation in the peripheral airways [27] which potentially increases the NO producing surface[15]. Another explanation is an increase in the diffusion of NO towards the lumen caused by epithelial damage[28,29], thickened basement membrane[28,29] and subepithelial fibrosis[30].

The lack of a relationship between allergic sensitisation and Calv_NO _suggests that no alveolar inflammation is caused by the IgE sensitisation per se. Allergic asthma has been "classically" reported as only having a bronchial inflammation component[16]. However, some recent studies report that symptomatic asthmatic patients [31,32] also have an alveolar component in the inflammation.

In the present study, exhaled NO levels and flow-independent airway NO-exchange parameters were related to the degree of allergic sensitisation. This was found both when assessing the level of sensitisation by the number of allergens and when adding the specific IgE titres. Franklin et al. [3] first reported the association between exhaled NO levels and the number of positive skin prick tests, an association that persisted even after adjusting for confounders. The association between exhaled NO levels and the skin prick test index was first reported by Barreto et al. [4]. Assessing the degree of sensitisation by adding the specific IgE titres was first proposed by Wickman et al. [8], who reported that, by using a combination of the number of positive allergens at test and the sum of specific IgE levels, it was possible to detect 90% of the individuals with an allergic disease. Syk et al. [9] reported that there was a relationship between the sum of specific IgE for perennial allergens (cat, dog, horse, mite and mould) and exhaled NO levels (r = 0.47). Total IgE may also be used to assess the degree of sensitisation and, in asthmatic children, Cardinale and co-workers[33] found a closer relationship between exhaled NO levels and total IgE (r = 0.42) than between the number of positive SPT and exhaled NO (r = 0.31). In the present study, the sum of specific IgE levels was more closely related to exhaled NO levels than total IgE levels. A similar finding was reported in a recent study[5] in which exhaled NO was more closely related to prick index (r = 0.37) than to total IgE levels (r = 0.22).

Sensitisation to cat allergen was the type of sensitisation that was most closely related to exhaled NO. This is in accordance with other studies showing that perennial allergens and not seasonal allergens are the main determinants of high exhaled NO levels [4,34,35]. There are, however, geographical differences regarding both sensitisation to different allergens[36] and the type of allergen that appears to play the most important role. In Southern Europe, mite sensitisation is the main determinant of increased exhaled NO levels[4], while in Northern Europe pet allergens (cat and dog) are the allergens that have the greatest impact on exhaled NO levels[6].

No differences in exhaled NO levels were found between cat-allergen-sensitised subjects who had or did not have a cat. This result is in accordance with a study of schoolchildren from the same geographical region[6], while studies using measured allergen exposure have produced conflicting results[37,38]. The lack of association between pet ownership and exhaled NO is probably related to the fact that cat allergen is widespread in Sweden and basically everybody is exposed to it in low doses [39].

Rhinitis and asthma were no longer independent determinants of exhaled NO after adjusting for the degree of IgE sensitisation. Our results are in line with recent studies[7,40] supporting the theory that the increase in FE_NO _values reported in allergic respiratory diseases are more due to the atopic status (IgE sensitisation) than to the respiratory disease per se. In contrast to this, an independent effect of both asthma and sensitisation to perennial allergens on FE_NO _was found in a recent Swedish study[41]. In this study of bleachery workers, no adjustment was, however, made for the degree of sensitisation. The results of a recent study [42] using exhaled NO to adjust the inhaled corticosteroid doses in the treatment of asthma do, however, support the notion that exhaled NO levels are related to some extent to the asthmatic inflammation and not only to the degree of IgE sensitisation.

The main problem when it comes to interpreting our results is the relatively low participation rate. There were, however, no significant differences between the participants and non-participants regarding IgE sensitisation and smoking and we therefore do not feel that this has affected our results to any marked degree. Another problem is related to the choice of allergens. Birch, one of the most common allergens in Sweden, was not included in the study. Previous studies examining the correlation between different IgE titres and NO have only found a weak correlation between IgE titres against birch and exhaled exhaled NO [6,9]. Monosensitisation to birch allergen is not so common and an analysis from the ECRHS I study [43] has confirmed that after excluding birch sensitisation there was only a modest decrease in the prevalence of IgE sensitisation. The definition of asthma in the present study was based on self reported diagnosed asthma in combination with asthma symptoms within the last 12 months. De Marco et al. [44] analyzed data from the three Italian ECRHS I centres and reported that this definition of asthma underestimated the actual number of subjects with clinical verified asthma. This could be one reason for the differences between our study and a recent study [45] that found that exhaled NO was a valuable method for diagnosing asthma in subjects with suggestive symptoms of asthma. It should, however, be noted that the prevalence of atopy in the cited study was 76% in those that were diagnosed as having asthma and 43% in the non-asthmatic group.

## Conclusion

IgE sensitisation is related to an increase in airway NO transfer factor and an increase in NO concentration in the airway wall. These NO variables were also positively related to the degree of allergic sensitisation. Sensitisation to cat allergen was related to the highest increases in exhaled NO levels. Our data suggest that exhaled NO is probably more a specific marker of allergic inflammation than a marker of asthmatic inflammation.

## Competing interests

The author(s) declare that they have no competing interests.

## Authors' contributions

CJ and MH designed the study. Data was collected by CJ, TH, PM and MH. The statistical analysis and data interpretation were performed by AM and CJ. The manuscript was prepared by AM and CJ. AM performed the literature search. AM, CJ, DN and MH have obtained the funds necessary to perform the study.

All authors have read and approved the final manuscript.
